# Bilateral Concurrent Benign Phyllodes Tumor in a 43-Year-Old Female: A Case Report

**DOI:** 10.7759/cureus.37588

**Published:** 2023-04-14

**Authors:** Andres Francisco, Jennifer Beniquez Martir, Jesus E Guarecuco Castillo, Rana El-Tawil, Mohammed M Masri

**Affiliations:** 1 General Surgery, Nova Southeastern University Dr. Kiran C. Patel College of Osteopathic Medicine, Fort Lauderdale, USA; 2 General Surgery, University of Medicine and Health Sciences, Camps, KNA; 3 General Surgery, Larkin Community Hospital, South Miami, USA

**Keywords:** fibroadenoma, fibroepithelial tumors, cystosarcoma phyllodes, bilateral phyllodes tumor, phyllodes tumor

## Abstract

Phyllodes tumor is considered a rare form of breast tissue neoplasm that presents as a rapidly growing painless mass. This neoplasm is classified as benign, borderline, or malignant and standard treatment consists of surgical excision with clear margins. The vast majority of reported cases have described the unilateral presentation of this tumor, making bilateral presentation a rare find. Our case describes a 43-year-old Hispanic woman with a history of fibroadenomas who was found to have concurrent benign bilateral phyllodes tumors.

## Introduction

Johannes Muller originally discovered the phyllodes tumor in 1838 and described the tumor as cystosarcoma phyllodes [1]. However, contrary to their original naming, further studies have shown these to be fibroepithelial tumors. This rare fibroepithelial tumor makes up 0.3% - 0.5% of all female breast cancers, with the incidence of bilateral synchronous cases being even rarer [1]. Typically, this tumor presents in females between the age of 40-50 years, with an average age of 45 years. It is described as a rapidly growing, painless well-circumscribed mobile mass with an average diameter of 4-5 cm [2,3]. These tumors can grow rapidly and may present with a biphasic growth pattern. As the tumor grows, a visible mass forms, distorting the breast’s contour. Larger tumors may cause visible skin changes on physical exam; skin can appear stretched and shiny. In rare cases, these tumors have been associated with nipple retraction, ulceration, and chest wall fixation [4].

Percutaneous core needle biopsy is required for histological diagnosis of these tumors. Sometimes, a diagnosis is made after an excisional biopsy of a presumed fibroadenoma [4]. Microscopically these tumors present with a characteristic leaf-like structure composed of elongated cleft-like spaces containing papillary projections of epithelial-lined stroma with varying degrees of atypia and hyperplasia [4]. Although most cases are considered benign, they may present on a spectrum from benign to malignant. The World Health Organization (WHO) classifies these into three categories: benign, borderline, and malignant. This classification is based on histological features, including stromal cellularity and overgrowth, nuclear atypia, mitotic activity, and margin appearance [5]. However, the National Comprehensive Cancer Network (NCCN) states that there is no uniform agreement concerning the criteria for classifying these subtypes [6]. The treatment for these tumors is wide surgical excision with margins greater than 1 cm to avoid the subsequent growth of any remnant tissue [7].

## Case presentation

**Figure 1:**
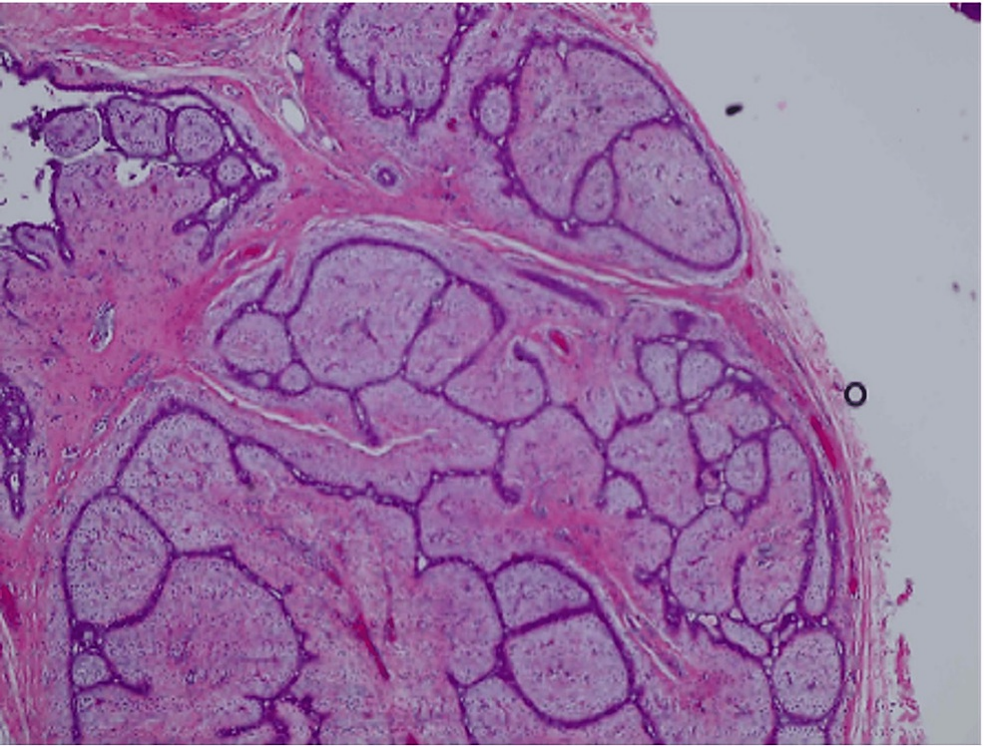
2.2 cm left breast mass- long lateral, short superior section showing a benign phyllodes tumor with positive margins and no atypia or malignancy.

**Figure 2:**
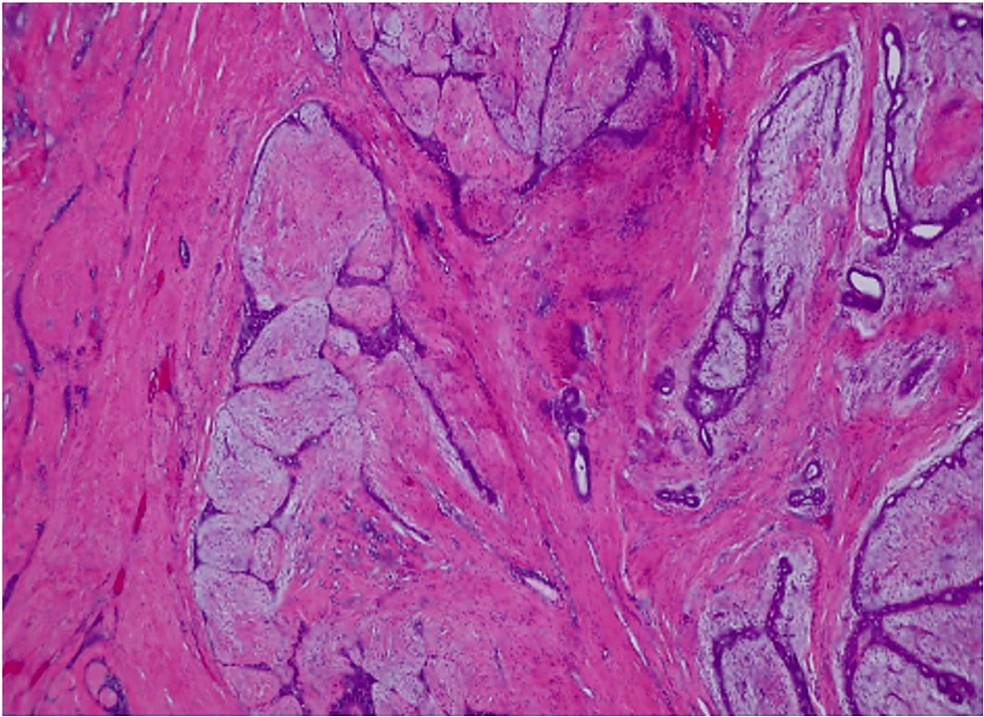
1.3 cm right breast mass showing a benign phyllodes tumor with positive margins and no atypia or malignancy.

**Figure 3:**
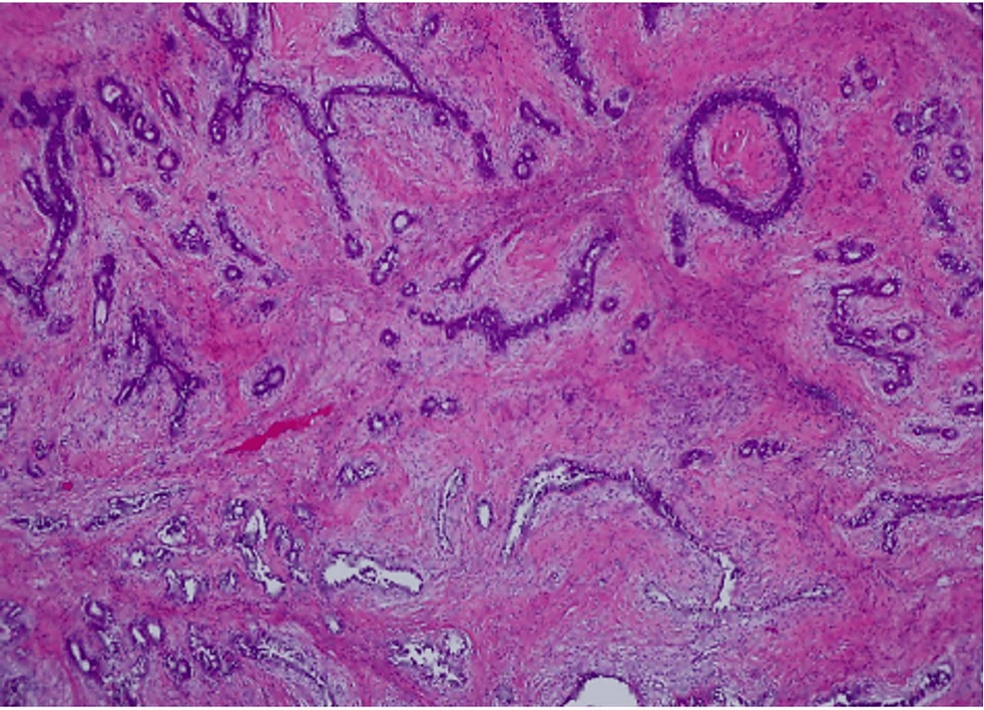
1.8 cm chest wall mass showing a benign phyllodes tumor with positive margins and no atypia or malignancy.

A 43-year-old Hispanic female with no significant medical history presented with a left breast lump with associated discomfort. She reported a positive family history of thyroid cancer in her mother. The patient’s personal history includes bilateral breast masses, which underwent bilateral lumpectomies in 1990 and 2015, with pathology showing fibroadenoma in both instances. Seven years after her last lumpectomy, bilateral isodense breast masses and minimal, benign-appearing calcifications were found on screening mammography. Her Breast Imaging Reporting and Data System (BI-RADS) score was determined to be zero, and further workup was needed. Ultrasound was performed and visualized multiple bilateral hypoechoic nodules that were thought to be fibroadenoma. A BI-RADS score of three was assigned to the masses based on the ultrasound findings, suggestive of benign findings but warranted additional surveillance. The patient presented to the clinic due to the discomfort caused by the bilateral nodules, which were palpable on physical exam. No swelling, dimpling, retractions, or nipple discharge were seen upon examination of the patient. The right 2 cm breast nodule was peri-areolar, and the left 2.8 cm nodule was retro-areolar at the one o’clock axis. The patient was offered ultrasound-guided biopsy but refused and preferred to undergo surgical treatment. A bilateral lumpectomy was performed promptly, and intraoperative samples were preserved from bilateral breast masses for pathological analysis (Figure 1-3). 

Pathology report characterized both the right and left breast masses as benign phyllodes tumors with positive margins and no sign of malignancy. Following the results of the pathology report, the patient decided to undergo surgical management to remove possible remnant phyllodes tumor. Approximately one week after the primary surgery, the patient underwent a bilateral quadrantectomy with wide-margin excision. The prior incisions were used, and dissection of the superior, inferior, lateral, medial, and deep margins were re-excised. Intraoperative samples were sent for pathologic analysis to confirm benign tissue with no concern of atypia. The final pathology report showed no significant residual phyllodes tumor and clear margins.

## Discussion

The benign synchronous presentation of phyllodes tumors is rare and poorly understood. In our literature review, only five cases of bilateral benign synchronous phyllodes tumors have been reported, with our case being the sixth [8-12]. Data collected in a separate article found that 22 out of 172 benign phyllodes tumor patients had reports of a previous fibroadenoma [13]. The authors theorized that the development of phyllodes tumors might be linked to the progression of prior or incomplete resection of a fibroadenoma [13]. Our patient further supports the theory of phyllodes tumor development from the progression of a fibroadenoma due to the past surgical history of two fibroadenoma removals. The article also found that 11 out of 36 patients with malignant phyllodes tumors had a history of fibroadenoma, emphasizing the importance of patient follow-up to catch these phyllodes tumors before they grow and metastasize [13].

Pathophysiological data among unilateral phyllodes tumors is scarce; thus, bilateral phyllodes data is near nonexistent, but the two may overlap. In 34 patients with phyllodes tumors who underwent genetic testing, approximately 10% of these patients had a deleterious mutation [14]. Further investigation is warranted into whether the genetic predisposition is the primary etiology of the bilateral cases of phyllodes tumor or other factors are responsible. One of the challenges faced in pinpointing a conclusive etiology is the small sample size of unilateral and bilateral phyllodes tumors.

## Conclusions

The amount of documented cases of bilateral phyllodes tumors is extremely low, which contributes to the lack of understanding about the mechanism or possibly precipitating factors. Our documentation of this case can contribute to a possible pattern of risk factors such as previous fibroadenoma and possible incomplete resection. Additional studies can focus on the differences in presentation, if any, between unilateral and bilateral phyllodes tumors and establish any pattern in patient history. The importance of documenting these cases remains high to reinforce present theories further and identify patients who warrant further surveillance based on their presentation. Our patient was managed appropriately with wide-margin excision and continues to follow up to monitor for abnormal growth or masses.
